# Impact of Non‐Uniform Standards in Forensic Postmortem Paediatric Microbiology on the Evidentiary Value of Microbiological Findings: A Scoping Review of Postmortem Sampling Sites, Techniques and Interpretation Methods

**DOI:** 10.1111/apm.70262

**Published:** 2026-09-14

**Authors:** Sarah Parsons, Judith Fronczek, Reena Sarkar

**Affiliations:** ^1^ Victorian Institute of Forensic Medicine Southbank Victoria Australia; ^2^ Department of Forensic Medicine Monash University Melbourne Victoria Australia

**Keywords:** autopsy, contamination, paediatric, postmortem, postmortem microbiology (PMM)

## Abstract

To map international literature on the extent, nature and standards of evidence on postmortem microbiology (PMM) sampling in paediatric cases. Investigations of sudden unexpected deaths in infancy and childhood warrant high standards due to their public health and legal significance. PMM is an integral component of these investigations. The review aimed to examine paediatric studies; however, only a single study was identified. The review therefore included articles on PMM of all ages and forensic and clinical autopsies. Data sources used were Medline, Embase, Cochrane CENTRAL and Scopus. Primary studies were selected by title and abstract screening using Covidence. Twenty four primary studies covering 2965 postmortems with an age range of 0–90+ years. There was significant heterogeneity in the selection of sampling sites and interpretation, with no consistent definition for contamination or true infection. There were significant gaps in literature, particularly the lack of paediatric studies, standardised techniques for specimen collection and definition of contamination. An interpretation framework for PMM results was proposed by the authors. These gaps warrant immediate attention as inconsistent interpretation of PMM results can lead to low public confidence in mortality statistics, disease surveillance data and public health policy.

**Abbreviations**
CSF: cerebrospinal fluid
JBI: Joanna Briggs Institute
PMI: postmortem interval
PMM: postmortem microbiology
SADS: Sudden Arrhythmic Death Syndrome
SIDS: sudden infant death syndrome
SUDC: Sudden Unexplained Death in Childhood

## 1 Introduction

Microbiology is an integral component of standardised postmortem practice. Postmortem microbiology (PMM) sampling involves the systematic collection of biological specimens at the time of postmortem for the purpose of subsequent analysis in the laboratory. Microbiological investigations identify infectious pathogens that have caused or contributed to death, differentiate infection from post‐mortem contamination or translocation, support determination of cause of death, correlate postmortem findings with clinical findings and inform public health surveillance [1, 2].

Sudden infant death syndrome (SIDS), Sudden Unexplained Death in Childhood (SUDC) and Sudden Arrhythmic Death Syndrome (SADS) are all types of sudden unexpected deaths that occur in previously healthy individuals. PMM is required under the current international guidelines for investigation and diagnosis of SIDS and is recommended in the investigation of SUDC and SADS [3]. However, interpretation of the PMM tests is challenging owing to false positives due to bacterial translocation, contamination and the native biome. Paediatric cases are unique in that PMM should be performed routinely, even when non‐accidental trauma is suspected to exclude underlying infections that may mimic the clinical or pathologic presentation of intentionally inflicted injury. Additionally, the potential for contamination must be considered, as false positive microbiological findings may adversely impact cause of death determination, thereby compromising both the evidentiary value of PMM results and accuracy of mortality statistics.

Whilst PMM has been in use for decades, there is an absence of unified consensus on sampling techniques, definition of contamination and contamination control procedures. A lack of sampling and reporting standards means that meaningful comparisons between institutions are not possible. A standardisation guideline from Europe in 2019 recommends a combination sampling technique whereby an antiseptic solution is first applied to the area to be sampled for PMM, followed by searing. Searing is a postmortem sampling technique in which a flame is used to heat a metallic object, typically a blade, to sterilise the surface of a solid organ by thermal destruction of contaminating microorganisms prior to specimen collection [4]. In contrast, other guidelines, including those from the Australasian College of Pathologists in 2021, do not specify collection technique or reporting parameters for paediatric deaths [5].

Krous and Byard [6] published an international standardised protocol for sudden infant death in 1995 outlining sample location/site. This protocol recommended tracheal and stool specimens for viral culture and blood and CSF for bacterial culture, are collected prior to postmortem. Liver, lung and myocardium sample collections are collected during the postmortem. Tests for fungi and mycobacterium are done at the discretion of the forensic pathologist. Since then, no further international guidelines or protocols have been published. Krous's guideline was endorsed by the National Association of Medical Examiners (NAME) (who have published alternate protocols in 2019 for use in the USA) and Society of Paediatric Pathology USA. None of these guidelines specify case eligibility criteria (e.g., age range) or manner of PMM sampling.

This scoping review focused on paediatric cases including sampling sites and techniques and interpretation methods. By reviewing past and current procedures, this paper aimed to provide a foundation for future standardisation studies for improving PMM reliability and interpretation nationally and internationally and optimise medicolegal death investigations.

Specific review questions were:

1. Which samples are advised for PMM in cases of sudden death particularly in paediatric cases?
2. What were the different sampling techniques recommended for PMM (e.g., sterile sampling or searing)?
3. Are specific microbiology techniques recommended in paediatric autopsies less than 3 years?
4. What strategies assist in minimising contamination during PMM sampling?
5. Did postmortem interval affect contamination rates?

## 2 Methods

### 2.1 Protocol and Registration

The proposed scoping review was conducted in accordance with the Joanna Briggs Institute (JBI) methodology for scoping reviews [7] and reported as per Preferred Reporting Items for Systematic reviews and Meta‐Analyses extension for Scoping Reviews [8]. A preliminary search of Medline, PROSPERO and Open Science Framework was conducted using keywords: forensic postmortem microbiology (PMM) and/or autopsy microbiology. No registered current or underway systematic reviews or scoping reviews on the topic were identified.

### 2.2 Eligibility Criteria

The inclusion and exclusion criteria are described in Table 1 as per population, concept and context.

**TABLE 1:** Inclusion and exclusion criteria for scoping review.

	Inclusion	Exclusion
Population	Deceased individuals who underwent human autopsy with microbiology including prematurely delivered babies Scope of paediatric focus: In a mixed population there was no limits placed on the extent of qualitative or quantitative information in the papers Data were reported separately where disaggregated paediatric‐specific results were reported	Animal studies, in vitro or laboratory only studies Studies with no microbiology sampling (for e.g., sampling was performed but for genetic testing purposes only) Still born or Fetal Death in Utero
Concept	Studies describing or evaluating: Post‐mortem microbiological sampling techniques Sampling sites Contamination control Interpretation	Studies focused solely: Thanatomicrobiome Microbial succession without evaluation of sampling technique Microbiology where the sampling method was not described or could not be inferred
Context	Forensic pathology Anatomical pathology Mortuary Hospital autopsy settings	Clinical microbiology Surgical or clinical sampling unrelated to autopsy practice Environmental contamination studies not linked to autopsy sampling

### 2.3 Selection Process

A preliminary keyword searching of Ovid MEDLINE identified wide variation in study periods, postmortem types and sampling sites, preventing statistical pooling and meta‐analysis, justifying a scoping review approach.

This scoping review evaluated English language publications with a variety of study designs: observational descriptive, cross sectional, case control, experimental or comparative studies, methodology/validation studies and case series with ≥ 3 cases. Only peer reviewed published papers were included. Single case reports (< 3 cases); conference abstracts without full text; editorials, opinion pieces, letters without original data; narrative reviews; and non‐peer reviewed preprints were excluded.

### 2.4 Search Strategy and Information Sources

An initial limited search of MEDLINE (PubMed) was undertaken to identify original articles on the topic. The text words contained in the titles and abstracts of relevant articles and the index terms used to describe the articles were used to develop a full search strategy for Ovid MEDLINE, Embase, Cochrane CENTRAL and Scopus (see Appendix S1 for search strategies). The search strategy, including all identified keywords and index terms, was interchangeably adapted for each included database and/or information source. There were no limits for study period.

### 2.5 Study/Source of Evidence Selection

Following the search, all identified citations were collated, uploaded and de‐duplicated in Covidence [9]. Fifty titles and abstracts were screened by all reviewers as per PRISMA ScR guidelines to achieve consensus [8]. A single reviewer assessed the remainder against the agreed inclusion criteria. Reasons for exclusion at the full text screening stage were recorded and reported.

### 2.6 Data Extraction

A Covidence extraction template was developed that encompassed study characteristics, population, postmortem setting, specimen location and sampling technique, outcomes including microbe classification and contamination rate, clinical history, allied test results, influencing factors (e.g., postmortem interval/PMI) and interpretation of results. Data extraction was performed by a single observer and verified by another author. The extraction tool was piloted for 2 cases and amended for scope to include free text, not classifiable elsewhere. A second reviewer independently completed a further 2 full extractions with consensus between the 2 reviewers prior to the remaining 18 papers (75%) extracted by a single reviewer. As sex does not influence the collection of PMM, it was not reported as an observed variable. Table 2 shows the criteria used to categorise and interpret study findings.

**TABLE 2:** Criteria used to categorise and interpret studies.

	Criteria for categorisation	Interpretation
Study characteristics	Design Country Population Year	Context
Sampling location	Blood culture, cerebrospinal fluid, Nasopharyngeal Aspirate, lung, liver, spleen, middle ears, myocardium	Diagnostic utility and rationale
Sampling technique	Searing Ethanol Sterile collections Other	Potential sources of contamination Methods to improve reliability
Outcomes	Contamination rate Microbiological classification Clinical history Allied tests: histology, multiple sampling sites showing same bacteria, use of biochemical tests	Diagnostic value: distinguishing infection from contamination
Influencing factors	Postmortem interval Bacterial translocation	Effect on microbiology recovery/contamination rates

### 2.7 Risk of Bias

The JBI guidelines for cross‐sectional and case‐control studies were used to assess quality of the studies. Twenty percent of cases were independently assessed for bias by 2 reviewers with zero disagreement.

## 3 Results

**FIGURE 1:**
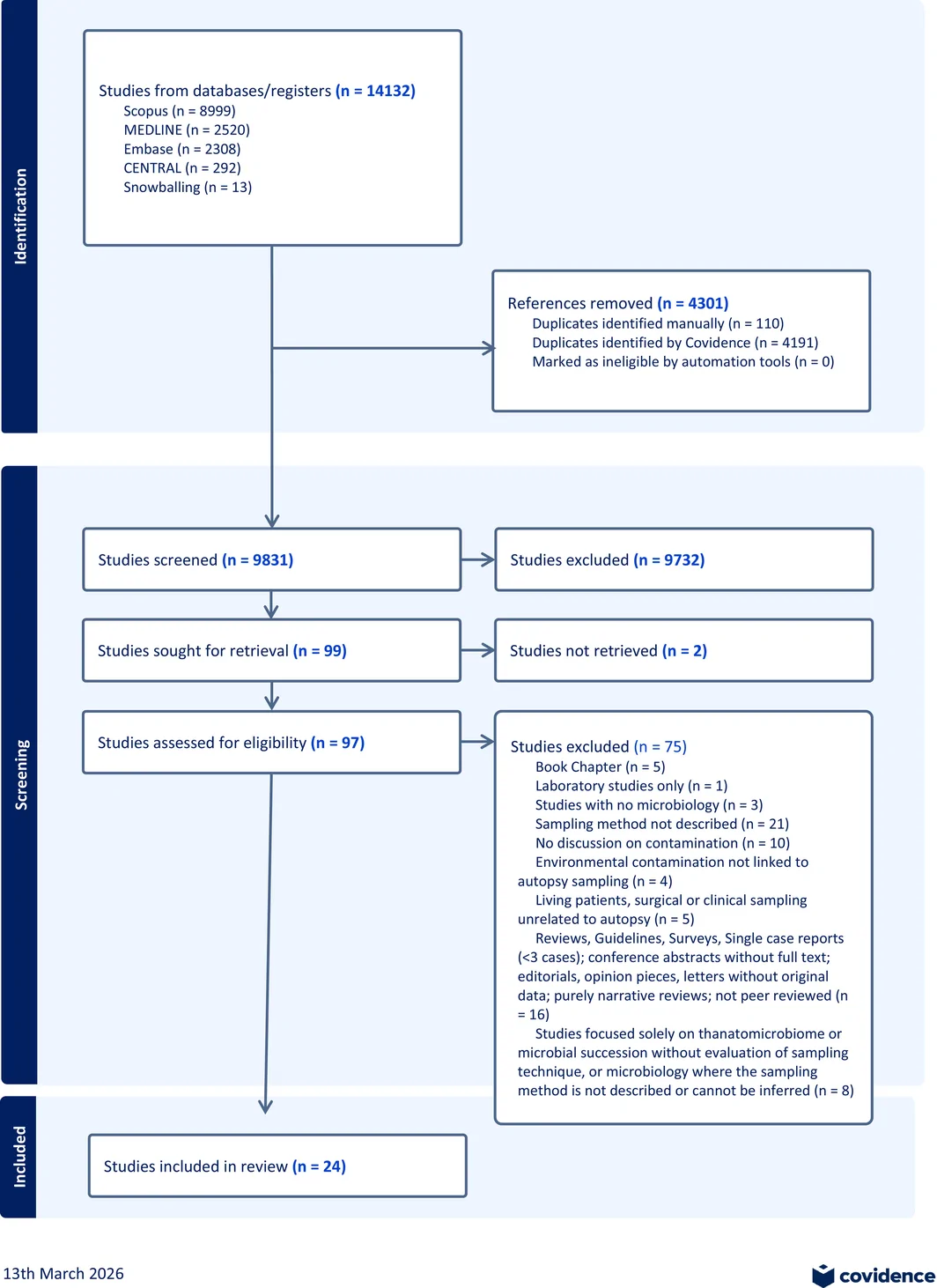
Microbiology sampling techniques during forensic autopsy and its correlation with contamination.

Twenty‐four papers were eligible for inclusion. The results of the search and the study inclusion process are reported comprehensively in a PRISMA flow chart (Figure 1) [10]. The review identified only one paper offering an exclusively paediatric focus of PMM concepts and techniques [11].

### 3.1 Paediatric Study Findings

The single paediatric Norwegian study included 305 cases autopsied under a local standardised protocol with age range: 7 days to 1 year (*n* = 181), newborns (*n* = 26), 1–3 years (*n* = 67) and > 3 years (*n* = 31). Blood and CSF samples taken in the hospital immediately after death and at the time of postmortem were compared. CSF was obtained at postmortem by a sub‐occipital puncture and (heart) blood after opening the thorax and pericardial sac by using sterile technique. Lung, liver, kidney and spleen were seared and sampled with a sterile knife and forceps. The median postmortem PMI was 24.25 h (95% CI: 22–25.5 h). The study found that no bacteria of diagnostic significance was found in hospital samples that could not be simultaneously traced in the postmortem samples. The authors concluded that the contamination rate was higher at postmortem owing to translocation of gastrointestinal flora after death. Only 4% of collected samples showed bacterial growth of diagnostic significance [11].

This study scored 9 on the JBI scale, indicative of a low risk of bias. The study demonstrated a rigorous methodology and provided a comprehensive and well‐developed study. As the solo paediatric study in the review, the study provided some evidence to answer research questions one on which samples to take in paediatric cases and four on postmortem interval. The study concluded that samples were stable and PMI had no influence on bacterial growth if the postmortem occurred within 48 h. Further samples of lungs, liver, kidney and spleen were taken at the time of postmortem. The authors concluded that blood cultures, CSF and lung tissue were the most informative samples for cause of death determination. They reported that sampling spleen, liver and kidneys added little independent information.

### 3.2 Study Characteristics

#### 3.2.1 Setting

Majority (70%) of the 24 primary studies (including one with paediatric focus) were hospital‐based. Of 24, 7 (29%) were forensic‐based (where a forensic centre was a specialised entity that performed postmortems to support medicolegal death investigations). All study reports were for single centres, which may not be representative of broader populations.

#### 3.2.2 Population

The studies were predominantly United States‐based, with a minority from Europe, China and Japan representing variable healthcare systems, medicolegal death investigation systems/frameworks, postmortem practices and personnel competencies governing the postmortem examinations. The study characteristics and risk of bias are described in Table 3.

**TABLE 3:** Study characteristics.

Lead author, year	Year country	Post mortem type	Study design	JBI risk of bias	Score
Giordano, 1921 [12]	USA	Hospital	Case series	Moderate	4
Epstein, 1929 [13]	USA	Hospital	Case series	Moderate	4
Burn, 1934 [14]	USA	Hospital	Case series	High	2
Kurtin, 1957 [15]	USA	Hospital	Comparison analysis	Low	8
Wood, 1965 [16]	USA	Hospital	Comparison analysis	Moderate	5
O′Toole, 1965 [17]	USA	Hospital	Case series	Low	8
Koneman, 1969 [18]	USA	Hospital	Comparison analysis	Low	7
Silver, 1969 [19, 20]	USA	Hospital	Comparative analysis	Low	8
Wilson, 1972 [21]	USA	Hospital	Comparison analysis	Low	8
Zanen‐Lim, 1980 [22]	Netherlands	Hospital	Case series	Moderate	6
Fung, 1983 [23]	USA	Hospital	Case series	Moderate	5
Wilson, 1993 [24]	USA	Hospital	Comparison analysis	Low	8
Søgaard 1990 [25]	Denmark	Forensic	Comparative analysis	Moderate	5
Lobmaier, 1994 [11]	Norway	Forensic	Comparative analysis	Low	9
Hove, 1998 [26]	USA	Hospital	Comparative analysis	Low	7
Palmiere, 2008 [27]	Switzerland	Forensic	Cross sectional	Low	7
Zheng, 2011 [28]	China	Forensic	Case series	Low	9
Tang, 2013 [29]	China	Hospital	Cross sectional	Low	8
Christoffersen, 2015 [30]	Denmark	Forensic	Case Series	Low	8
Sunagawa, 2017 [31]	Japan	Hospital	Comparison analysis	Low	7
Spagnolo, 2019 [32]	Italy	Forensic	Case series	Low	8
McMullen, 2019 [33]	USA	Hospital	Comparative analysis	Moderate	6
Tambuzzi, 2022 [34]	Italy	Forensic	Case series	Low	8
Diac, 2022 [35]	Romania	Forensic; hospital	Cross sectional	Low	8

*Note:* Risk of bias based on JBI critical appraisal checklist: case series—0–3 low quality/high risk of bias, 4–6 moderate quality/moderate risk of bias, 7–10 high quality/low risk of bias. For analytical cross section studies—0–3 low quality/high risk of bias, 4–6 moderate quality/moderate risk of bias, 7–8 high quality/low risk of bias.

Abbreviation: JBI, Joanna Briggs Institute.

The total number of postmortem cases examined across 24 studies was 2965, ages ranging from 0 days (still born) to 90+ years. Three papers examining Printculture reported on samples taken from selective organs only (lung and spleen) [22, 23, 33]. Printculture is a method of microbiology analysis which involves cryostat mounted sections of frozen tissues imprinted onto agar surfaces. Although paediatric populations were included in the mixed age studies, there was insufficient age‐specific disaggregation for microbiology techniques.

As previously stated, only one study exclusively examined paediatric cases aged 7 days to 3 years [11]. Nine studies had mixed age group cases, three examined exclusively adult populations and eleven studies did not document age.

### 3.3 Risk of Bias

Risk of bias was assessed with the JBI Critical Appraisal checklist for cross‐sectional and case series studies [36]. The predominant study design was case series in fourteen (58%) studies. One case had a high risk of bias given inconsistency between postmortems and no inclusion or exclusion criteria or demographic data. Nonetheless, this pilot study of single pathologist cases had an interesting discussion on contamination and normal flora and was deemed eligible for the review.

A high or moderate risk was observed in eight cases; five case series and three comparative analyses. The remaining sixteen low‐risk (scores between 7 and 9) studies: five case series, three cross‐sectional and eight comparative analyses had clear inclusion and exclusion criteria, with consistent techniques and statistical analyses.

### 3.4 Sampling Location

There was heterogeneity of anatomic sampling sites in the studies with no consistent criteria documented for sampling site selection (Table 4). Six (25%) studies exclusively tested blood cultures. The remaining studies sampled a wide range of anatomic sites including liver, spleen, cerebrospinal fluid, myocardium, lung and kidneys. No studies showed direct comparison between sampling sites.

**TABLE 4:** Sampling techniques, location, postmortem interval, contamination rates, postmortem microbiology positive rates.

Author name, year	Cases (*n*)	Comparison group (*n*)	Sampling technique	Sample location	Postmortem interval	Contamination rate	Postmortem microbiology positive rate^a^
Giordano, USA [12]	213	0	Searing	Blood culture, spleen	1–23 h	NS	Blood—38% Spleen—39%
Epstein, USA [13]	66	0	Searing	Blood culture, myocardium, heart valve, bone marrow	0.08–27 h	NS	Blood—79% Bone marrow—100%
Burn, USA [14]	127	0	Searing	Blood culture, lung, liver, spleen, bowel, fluids, kidneys, bronchi, trachea, throat, brain, urine.	1–48 h	NS	87%
Kurtin, USA [15]	50	50	Searing	Blood culture, lung, spleen, peritoneum	2 periods: 12 h and 24–48 h	NS	26%
Wood, USA [16]	62	62	Searing	Blood culture	1–48 h	NS	83%
O′Toole, USA [17]	54	0	Sterile technique	Spleen, pancreas, kidney, psoas, lung	4–8 days	16%	54%
Koneman, USA [18]	91	56	Searing	Blood culture, lungs, liver and kidney	1–23 h	NS	In cases with clinical infection—Blood 39% Lungs 63% Liver 35% Kidney 47%
Silver, USA [19, 20]	325	325	4 methods^b^	Blood culture	1–25 h	NS	13% of all cases had clinically significant growth
Wilson, USA [21]	142	142	Searing	Blood culture, lung, liver, spleen	0–24 h	NS	Variable results depending on clinical group
Zanen‐Lim, Netherlands [22]	100	0	Nil	Lung	N/A	NS	24% dense growth, 43% patchy growth
Fung, USA [23]	65 spleen, 73 lung	0	Searing	Lung, spleen	N/A	NS	Spleen—55% Lung 35%
Wilson, USA [24]	148	111	Searing	Blood culture	6–66 h	62%	Single organism 6%; different organisms 2%; polymicrobial flora 9%
Søgaard Denmark [25]	42	10	Sterile technique	Lung, spleen	N/A	NS	36% of swabs showed full agreement with Printculture
Lobmaier, Norway [11]	305	437	Searing	Blood culture, CSF at 2 time periods after death. Lung, liver, kidney and spleen taken at postmortem	22–25.5 h	NS	Blood cultures—67% CSF—31.5%
Hove, USA [26]	149	149	Searing, iodine‐subclavian technique	Blood culture	Mean PMI (prospective) 12.6 h	NS	NS
Palmiere, Switzerland [27]	100	0	Searing	Blood culture, lung, liver, spleen	6–66 h	22%	35% of cases are positive in infectious cases. 19% positive unrelated to infection
Zheng, China [28]	131	0	Ethanol	Blood culture	4–48 h	4.50%	75.90%
Tang, China [29]	76	0	Ethanol	Blood culture	3–48 h	NS	66.6% positive
Christoffersen, Denmark [30]	42	0	Ethanol	Blood, culture, lung and as clinically indicated. In SUDI cases blood, lungs, CSF and Urine	NS	38% not relevant to cause of death	85.7% of cases are positive
Sunagawa, Japan [31]	31	31	Alcohol followed by 10% povidone iodine	Blood culture	1.5–18 h	NS	58.1%
Spagnolo, Italy [32]	36	0	Sterile	Blood culture, lung, liver, spleen, bowel, fluids, kidneys, bronchi, trachea, throat, brain, urine	24–28 h	5.50%	33.3% of samples positive
McMullen, USA [33]	13 postmortem, 5 surgical	18	None	Lung, pectoral muscle	≤ 72 h	3 cases showed a pattern consistent with contamination	9 cases were negative
Tambuzzi, Italy [34]	45	0	Sterile	Extensive list	< 48 h	NS	In infective cases 77% cultured a single bacterium, 11.5% fungus and 11.5% were mixed growth
Diac, Romania [35]	630	0	None	Blood culture, tracheal swab, Peritoneal fluid (most common extensive list)	1–8 h	NS	Infection was listed as COD in 83.2% of cases with positive microbiology

Abbreviation: NS, not stated.

^a^ Postmortem microbiology positive rates have inconsistent reporting methods.

^b^ Four methods in Silver et al. [19, 20] compared four different techniques of blood and tissue cultures by searing: (1) removing the sternum and sigmoid colon, then searing the atria (*n* 77, 24%), (2) the same method but leaving the bowel intact (*n* 84, 26%), (3) reflecting the skin and removing the intestine prior to searing the tissue using a needle to access the heart through the chest wall (*n* 82, 25%), (4) only reflecting the skin without manipulating the bowel (*n* 82, 25%).

### 3.5 Sampling Technique

Earlier studies from the 1920s through to the 1990s concentrated on variations of searing collection techniques. All fourteen studies prior to 1994 included this technique. Post‐1994, the studies examined a variety of non‐searing methods, such as sterile techniques and ethanol usage.

One in four studies exclusively employed searing technique for sampling (59% of 2939 postmortems). One United States study was conducted in a purpose‐built mortuary using surgical sterile procedures for sample collection. This would have significantly increased the cost and time required for PMM but ensured the methodologic rigour of the study [17]. The methodology was implemented only once and was not replicated in routine mortuary settings.

Two studies from the USA compared the differences in microbiologic yield and diagnostic significance between sampling methods. Silver et al. [19, 20] described experiencing a higher contamination rate in cases where the bowel was manipulated prior to PMM sample collection as compared to cases where the abdominal fascia was left intact prior to sampling. Hove and Pencil [26] described sampling blood from the subclavian vein that involved cleaning of skin with an acetone‐alcohol pad and subsequent scrubbing with a 10% povidone–iodine pad prior to aspirating blood. Those results were compared with the traditional searing technique of the atrium prior to collecting blood from the cardiac chamber at postmortem. The Iodine Subclavian method had a statistically significantly lower incidence of false positives than the searing technique (*p* = 0.035). The papers did not imply that one sampling technique was superior to another but recommended that local standards be used.

### 3.6 Outcomes

The outcome measured (i.e., case type or sample type) across the papers was not consistent, limiting the ability for direct comparison between studies. Due to significantly different methodology of the studies, we were unable to pool and interpret results.

#### 3.6.1 Contamination

**FIGURE 2:**
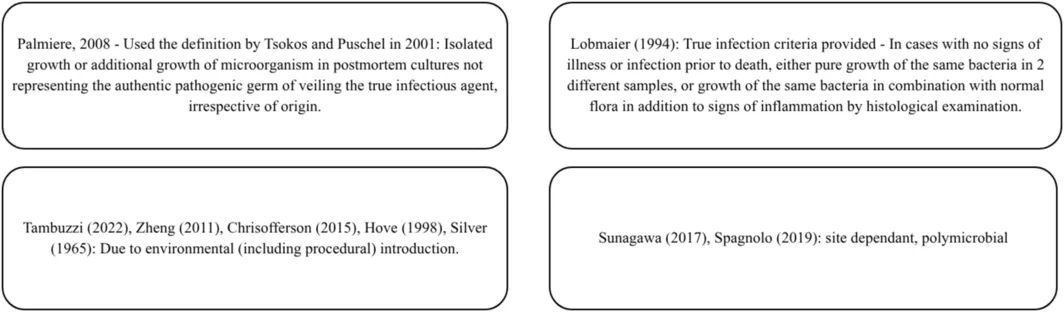
Patterns in review papers (*n* = 10) indicating inconsistency in contamination definition.

There was wide variation in contamination definitions and manner of reporting, limiting the generalisability for distinguishing contaminations from true infections. Five papers (21%) reported contamination proportions ranging 4.5%–62% (see Table 4). Of the 24 studies, only 9 provided some form of definition. Figure 2 summarises the four patterns adopted by studies in defining contamination. Table 5 highlights verbatim quotes to exemplify contamination definitions in the studies. It was generally agreed that contamination can be decreased with strict adherence to guidelines and maintaining aseptic conditions, regardless of methods of sampling. Three studies reviewed Printculture. The studies demonstrated that characteristic growth patterns of bacteria on agar plates were typical of contamination and true infection [23].

**TABLE 5:** Definition of contamination used in papers.

Study	Definition of contamination
Silver [20]	Contamination of cultures by the environment and by the personnel of the autopsy room
Lobmaier [11]	In cases with no signs of illness or infection prior to death, either pure growth of the same bacteria in two different samples, or growth of the same bacteria in combination with normal flora in addition to signs of inflammation by the histological examination, was required to deem it pathological and not contamination
Hove [26]	None provided—comment that contamination most commonly is due to lack of sterility in the sample technique
Palmiere [27]	Agree with the definition of Tsokos and Puschel ‘The isolated growth or additional growth of microorganisms in postmortem cultures not representing the authentic pathogenetic germ or veiling the true infectious agent, irrespective of origin’
Zheng [28]	Contamination: microorganisms come into samples at the time of collecting in autopsy
Christoffersen [30]	Contamination: Microorganisms are introduced into the tissue at time of sampling and were not present in life
Sunagawa [31]	Contamination: The contamination rate is influenced by the anatomical site, the skill of the clinician that collects the culture samples and the methods used. Most contamination occurs from the fluid found in body cavities and on organ surfaces. Contaminated culture samples can often be recognised because they exhibit polymicrobial growth that reflects the source of the contamination
Spagnolo [32]	Microbes are isolated from organs and fluids, which are normally sterile during one's life, the cultural result can be interpreted as contamination
Tambuzzi [34]	Bacteria accidentally introduced into samples obtained using instruments or operating in non‐sterile environments
Diac [35]	Contamination during the sampling procedures was characterised by the presence of a single or multiple microorganisms, frequently *Staphylococcus epidermidis* or another coagulase negative Staphylococci, alone or in association with commensals of the skin/mucosal membranes/mouth/upper respiratory tract

#### 3.6.2 Microbe Classification

Classification of microorganisms was inconsistent across studies. Microbes were classified by taxonomy, gram stain morphology and clinical relevance of the isolates (pathogenic/contamination/postmortem translocation).

#### 3.6.3 Interpretation of Findings

None of the studies explicitly referenced consistent regional or international guidelines governing interpretation of positive results. Some used local guidelines indicating internal validity between cases. Eleven studies with comparative analytic design measured a range of exposures such as antemortem/postmortem techniques, postmortem intervals and postmortem methods. A single positive culture was widely regarded as insufficient to establish infection. Many of the studies advocated positive cultures from multiple anatomic sites or correlating histology to aid in distinguishing true infection from contamination. A few studies highlighted poor correlation between antemortem and postmortem cultures, largely attributable to contamination issues.

#### 3.6.4 Allied Tests

Biochemical tests and histology were undertaken to support the diagnostic significance of PMM. The use of biochemical tests such as C‐Reactive protein and Procalcitonin, biomarkers of inflammation, were discussed in three papers. In summary, these tests were not reported as useful in supporting the PMM diagnosis of true infections as causes of death.

Histological findings were reported in three papers. These three author groups agreed that histology was an important way to distinguish contamination from true infection. They recommended PMM be considered contamination in the absence of corroborating histological evidence [25, 27, 30].

#### 3.6.5 Clinical History

Clinical history was variably documented in the papers. However, cases were categorised according to clinical presentation such as sudden death or infectious causes.

### 3.7 Influencing Factors

PMI (ranging 1–72 h) was documented in 20 (83%) of the 24 studies. The influence of extended PMIs (> 72 h), often observed in forensic practices, was not reported. Postmortem intervals ≤ 72 h did not influence contamination. One paper suggested that temperature‐sensitive bacteria may not grow after a period of refrigeration leading to false‐negative results for some bacteria [11].

## 4 Discussion

A scoping review was conducted to identify best practice in post‐mortem microbiology sampling techniques and locations, with the aim of mapping current practices to existing standards, particularly in paediatric forensic settings. Significant variability and lack of standardisation in PMM contribute to its diagnostic uncertainty in cause of death determination. Multiple strategies have been proposed in the literature to minimise contamination, including acquiring samples as close to the time of death as possible, immediate refrigeration of the deceased body, using a heated sterile blade to sear the organ surface (searing) and use of sterile instruments. There was no routine set of sampling locations that were consistently recommended in cases of sudden death in the papers examined nor was a single collection technique preferred over another for minimising contamination. No specific techniques were recommended for the paediatric age group < 3 years, in these postmortems. A postmortem interval of less than 72 h does not influence results if the body is refrigerated close to death.

### 4.1 Proposed Framework

**FIGURE 3:**
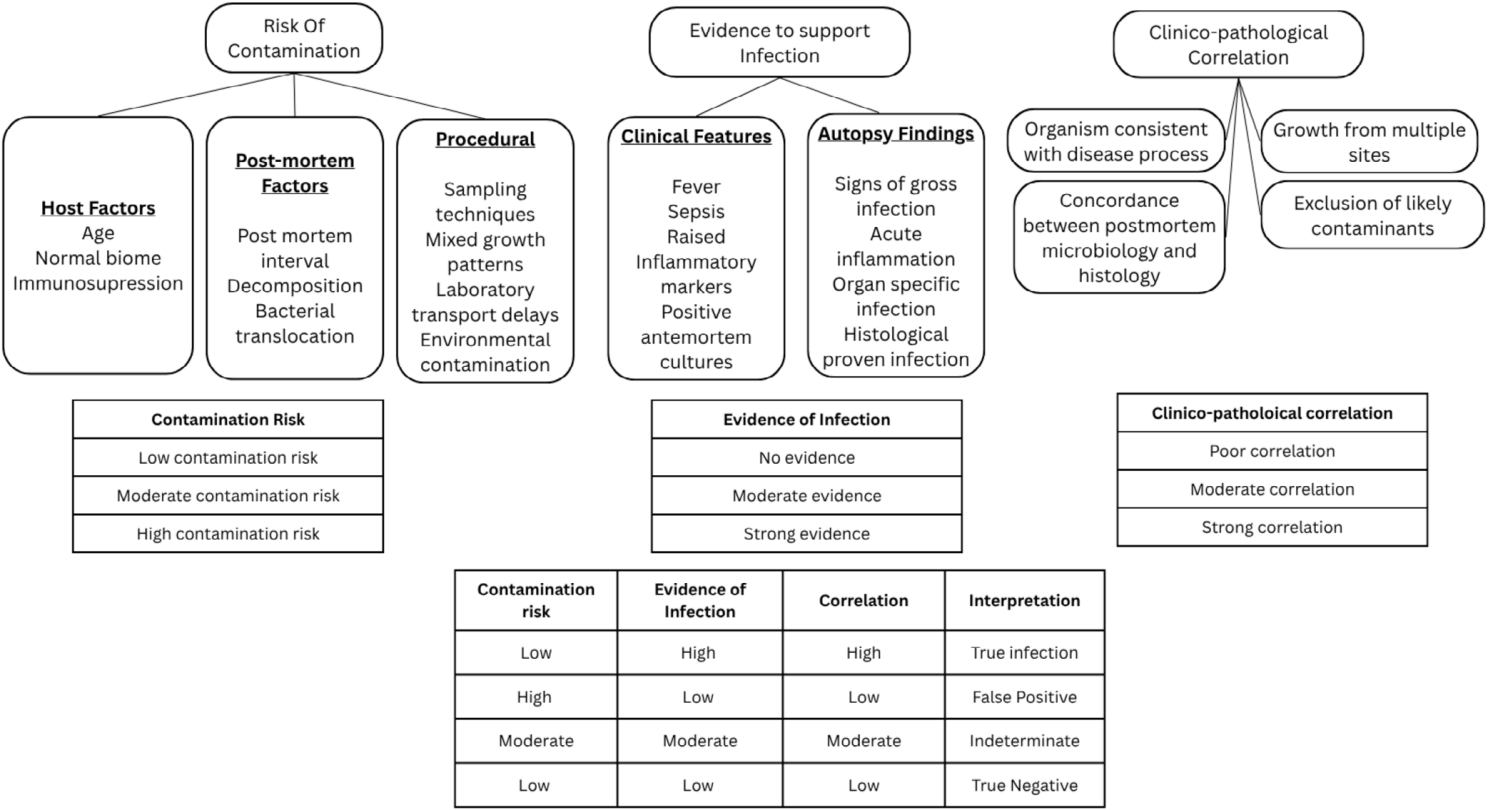
Framework for postmortem microbiology interpretation.

The authors propose a novel framework for interpreting postmortem microbiology and contamination mitigating the influence of and adjusting for effect modifiers in Figure 3. It works by assigning ordinal scores (low, moderate, high) to three key pillars: contamination risk, evidence of infection and clinico‐pathological correlation. The risk of contamination is divided under 3 subheadings: host factors, postmortem factors and procedural issues. Numerical scores have not been used in the proposed framework as each case requires adjudication by the forensic pathologist. We believe that the gold standard for true infection is histologically confirmed infection.

### 4.2 Principle Findings

1. A lack of unified definition for contamination is a key barrier to reproducibility.
2. Postmortem interval < 72 h does not influence contamination rates.
3. No standardised recommendations for which samples to take are available for PMM.
4. Searing was the most studied method for taking samples prior to 1994. After this period, sterile techniques with or without the use of ethanol or other sterilising fluid were identified. No technique has been recommended over the other.
5. The studies identified the absence of a standardised framework for PMM interpretation, particularly those specifying the distinction between true‐positive results and contamination.

### 4.3 Contributors of Variability in Postmortem Microbiology Interpretation and Contamination

The variability observed in postmortem microbiology findings was multifactorial, reflecting differences in mortuary practices such as sampling techniques and body location and PMI. Variation in mortuary setup and training processes is likely a procedural influence governing contamination rates, microbiologic yield and interpretations. This is further compounded by the inconsistency in definitions of contamination and what constitutes a true infection.

Though most studies examined blood culture, there was no consistent method for interpreting positive results. There was also a large gap in the literature on organ (spleen, liver and lungs) sampling as well as sampling comparisons between organs and blood/cerebrospinal fluid.

Regardless of technique, contamination issues remain similar throughout the decades and techniques did not change significantly, with no technique being preferred over another. Proposed European Guidelines from 2019 for PMM sampling at postmortem by a large multidisciplinary team of experts on microbiology and autopsies (both forensic and clinical) recommended either aseptic technique and/or searing for sample acquisition. These recommendations were based on a literature search and the group's own personal experience and expertise [35].

Comparisons between sampling techniques suggested less invasive sampling performed under strict aseptic conditions before manipulation of the bowel may reduce contamination or false positive results.

The papers examining Print culture technique demonstrated different growth patterns between true infection and contamination. This method, whilst used clinically, is not used in the forensic setting.

The differentiation between contamination and bacterial translocation is not straightforward. A key conceptual problem is the lack of accepted criteria to distinguish true infection and contamination. Agonal spread, postmortem translocation along with environmental or procedural contamination limit diagnostic certainty and reduce PMM contribution in cause of death determination.

One of the first attempts at standardising the terminology for contamination was made by Tsokos and Püschel [37] who defined contamination as the ‘isolated growth or additional growth of microorganisms in postmortem cultures not representing the authentic pathogenic germ or veiling the true infectious agent, irrespective of origin’. That review article stated contamination during (or after) sampling procedures was not systematically due to inadequate sampling procedures and that the anatomy of the investigation site (i.e., deeply located organs) and transient and/or resident bacterial colonisation of skin, mucosal surfaces, internal organs, or body fluids prior to death might be responsible for bacterial contamination.

Other researchers conceded that contamination was inevitable, given the presence of native biomes, bacteria involved in decomposition and bacterial translocation after death. All the studies reviewed highlight the issue of contamination and interpretation. Other researchers with whom the authors agree recommended the interpretation of PMM in conjunction with histological and/or clinical correlation. Positive PMM alone could not be relied on as a true clinical infection or relevant to cause of death. In 1990 Søgaard et al. [25] concluded: ‘The question of infection in vivo v. agonal bacterial invasion v. postmortem dissemination can never be solved by culture regardless of quantitation but should be answered by the presence or absence in the tissue of an acute inflammatory response found by histological examination’.

In conclusion, we agree with Epstein and Kugel [13] statement: The question of whether bacterial findings represent an agonal or postmortem invasion can only be settled by further studies. Since there is an absence of a generic list of ‘pathogenic’ bacteria or ‘contamination’ bacteria, it is implied that the available ancillary data needs to be interpreted on a case‐to‐case basis. Fungal and viral infections have not been considered in this review.

### 4.4 Paediatric Focus

Whilst PMM is essential in paediatric autopsies in infants and young children aged 3 and under, there is a distinct paucity of studies in this population, with only a single study identified. However, there was no difference in the sampling techniques in adult and paediatric PMM.

### 4.5 Universal Nature of Contamination and Sampling Techniques

The same underlying biological processes occurred after death regardless of age group. Contamination occurred when microorganisms were introduced at the time of sampling, such as commensal on the skin being transferred to blood culture that were not present in life. Sampling techniques such as searing, aseptic, or ethanol use are not age specific. This is highlighted by the fact that most of the studies (46%) did not report on the age of the deceased. In both adults and children, PMM results can either represent a true infection, postmortem translocation, contamination, or colonisation. Standard laboratory methods are universal and thus, in terms of sampling techniques and contamination, the results are likely replicable across age groups. Notwithstanding, the relevance of PMM in determination of cause of death across ages is outside the remit of the current paper.

The findings of the only paediatric study suggest that sampling of body regions: blood, CSF and lung have the maximum diagnostic yield provided sampling performed within 48 h [11]. These conclusions, however, are based on a single study and are non‐generalisable. This differs from the Krous criteria and UK criteria that recommend at least six different samples of body fluids and solid organs [6]. The more streamlined approach recommended by Lobmaier is likely to be embraced by pathologists over other criteria.

### 4.6 Implication for Practice

There is a need for clearly defined sampling sites, sampling techniques and consistent reporting of contamination for all mortuary standard operating practice. Improved procedural standardisation at the time of postmortem will also assist with mortuary technician training, diagnostic reliability and reduce interpretive uncertainty in cause of death determination and optimisation of medicolegal best practices.

### 4.7 Limitations

The study has some limitations. Although a comprehensive search strategy was employed across multiple databases, it is possible some studies were not identified due to methodological limitations, such as language restrictions and the exclusion of grey literature.

The included studies demonstrated considerable heterogenicity in study design, populations, methodology and definitions of contamination. This limits direct comparison across the studies and precluded quantitative synthesis. Although a framework integrating histological data and postmortem findings has been proposed, future research is needed for validation that may eventually support the diagnostic validity of postmortem microbiology.

Only English literature was included which may miss relevant research published in other languages, meaning findings may not fully represent findings and practices in non‐English speaking countries. The lack of low and middle‐income countries represented in the studies means the applicability of PMM is not representative. A number of these countries, especially in Africia, predominately utilise verbal autopsies and minimally invasive autopsy such as needle biopsies to collect microbiology samples [38].

The use of a single reviewer for screening and data extraction introduces a potential source of bias. This has been minimised by 50 full text screenings done by all 3 authors and 25% of cases having data extraction by 2 authors.

## 5 Conclusions

This scoping review mapped international literature on the extent, nature and standards on post‐mortem microbiology (PMM) sampling and interpretation. The review demonstrated major gaps in literature, particularly the lack of standardised collection techniques and definition of contamination. Only a single study examined a paediatric population under the age of three. This is concerning as PMM is mandated or recommended in sudden and unexpected deaths in infants and children. These gaps can significantly undermine the relevance of PMM results and public confidence in mortality statistics, disease surveillance data and public health policy.

We recommend correlation with clinical features and histological findings to assist in determining the utility of postmortem microbiology findings and their potential relevance in cause of death determination. A unifying framework is proposed as a structured approach for integrating microbiology findings with other factors.

## Funding

This review contributes towards Dr. Parsons PhD thesis. This research was supported by the Commonwealth through an Australian Government Research Training Program Scholarship [DOI: https://doi.org/10.82133/C42F‐K220].

## Disclosure

AI has not been used in the production of this manuscript.

## Conflicts of Interest

The authors declare no conflicts of interest.

## Supporting information


**Appendix S1:** Search strategy—scoping review.

## Data Availability

No data used to support this study.
