# Supplementary material for: Impact of Non‐Uniform Standards in Forensic Postmortem Paediatric Microbiology on the Evidentiary Value of Microbiological Findings: A Scoping Review of Postmortem Sampling Sites, Techniques and Interpretation Methods
Source: APMIS. 2026 Sep 14;134(9):e70262. doi: 10.1111/apm.70262 (PMC13573654; doi:10.1111/apm.70262)
Supplement: Supplementary file 1 [file APM-134-0-s001.docx]

**SUPPLEMENTARY MATERIALS 1: Search Strategy – Scoping Review**

**Database:** **Cochrane Controlled Register of Trials (CENTRAL)**

**Platform: Wiley**

**Publication Years: 1960 onwards**

**Date draft search was conducted on: 29^th^ January 2026**

**Limits: English Language Only**

**Filters: n/a**

**Yield Results: 292**

**Search Strategy:**

ID	Search	Hits

#1	[mh ^Autopsy]	76

#2	[mh ^"Postmortem Changes"]	7

#3	[mh ^Cadaver]	973

#4	autops*:ti,ab	677

#5	(postmortem*:ti,ab OR post-mortem*:ti,ab)	452

#6	(posthumous*:ti,ab OR post-humous*:ti,ab)	4

#7	cadaver*:ti,ab	2615

#8	necrops*:ti,ab	85

#9	death:ti,ab	82102

#10	#1 OR #2 OR #3 OR #4 OR #5 OR #6 OR #7 OR #8 OR #9	85607

#11	[mh "Forensic Microbiology"]	0

#12	[mh ^"Microbiological Techniques"]	58

#13	[mh ^"Bacteriological Techniques"]	316

#14	[mh ^"Molecular Diagnostic Techniques"]	131

#15	[mh ^"Diagnostic Tests, Routine"]	377

#16	[mh ^"Specimen Handling"]	874

#17	[mh ^"Blood Specimen Collection"]	493

#18	[mh ^"Urine Specimen Collection"]	50

#19	[mh ^Dissection]	356

#20	[mh ^"Blood Culture"]	52

#21	[mh ^"Viral Load"]	3448

#22	[mh "Viral Pseudotyping"]	0

#23	[mh ^"Virus Cultivation"]	44

#24	[mh ^"Virus Inactivation"]	11

#25	[mh Xenodiagnosis]	1

#26	[mh ^"Hot Temperature"]	2627

#27	("forensic" NEXT microbiol*):ti,ab	0

#28	("molecular" NEXT technique*):ti,ab	95

#29	("molecular" NEXT test*):ti,ab	456

#30	(specimen* NEXT "handling"):ti,ab	16

#31	(sear:ti,ab OR sears:ti,ab OR searing:ti,ab)	85

#32	cauter*:ti,ab	779

#33	swabbing*:ti,ab	205

#34	("blood" NEXT culture*):ti,ab	2004

#35	("blood" NEXT specimen*):ti,ab	636

#36	("urine" NEXT specimen*):ti,ab	609

#37	("specimen" NEXT collect*):ti,ab	410

#38	("sterile" NEXT technique*):ti,ab	188

#39	("aseptic" NEXT technique*):ti,ab	268

#40	("high" NEXT temperature*):ti,ab	328

#41	(heated:ti,ab NEAR/1 blade*:ti,ab)	0

#42	xenodiagnosis*:ti,ab	11

#43	#11 OR #12 OR #13 OR #14 OR #15 OR #16 OR #17 OR #18 OR #19 OR #20 OR #21 OR #22 OR #23 OR #24 OR #25 OR #26 OR #27 OR #28 OR #29 OR #30 OR #31 OR #32 OR #33 OR #34 OR #35 OR #36 OR #37 OR #38 OR #39 OR #40 OR #41 OR #42	14328

#44	[mh ^Infections]	4530

#45	[mh ^"Bacterial Infections"]	3735

#46	[mh ^Bacteremia]	995

#47	[mh ^Bacteriuria]	572

#48	[mh ^Microbiota]	676

#49	contaminat*:ti,ab	6255

#50	infecti*:ti,ab	130404

#51	pathogen*:ti,ab	22487

#52	#44 OR #45 OR #46 OR #47 OR #48 OR #49 OR #50 OR #51	153196

#53	#10 AND #43 AND #52 AND Limited to Trials AND English Language	292

**Database: Embase (last upload update 27^th^ January 2026)**

Platform: Ovid

Publication Years: 1946 onwards

Date draft search was conducted on: 29^th^ January 2026

Limits: English Language Only

Embase Database Only

Filters: Human Studies Only Filter

Yield Results: 2308

Ovid Medline Persistent Search Link (via Monash University Library):

<https://access.ovid.com/custom/redirector/index.html?dest=https://go.openathens.net/redirector/www.monash.edu?url=http://ovidsp.ovid.com/ovidweb.cgi?T=JS&NEWS=N&PAGE=main&SHAREDSEARCHID=3dLZCJdjRglpZ37DraUgG3rJ2nnOrcEyOy53cNuxZKRofnHbxYasgHI1Gi2HbJMaH>

**Search Strategy:**

| **#** | **Searches** | **Results** |
| --- | --- | --- |
| 1 | *Autopsy/ | 22403 |
| 2 | *Forensic Autopsy/ | 136 |
| 3 | *Postmortem Change/ | 1127 |
| 4 | *Cadaver/ | 8944 |
| 5 | autops*.ti,ab. | 144226 |
| 6 | (postmortem* or post-mortem*).ti,ab. | 111599 |
| 7 | (posthumous* or post-humous*).ti,ab. | 1136 |
| 8 | cadaver*.ti,ab. | 99995 |
| 9 | necrops*.ti,ab. | 37387 |
| 10 | death.ti,ab. | 1532589 |
| 11 | 1 or 2 or 3 or 4 or 5 or 6 or 7 or 8 or 9 or 10 | 1847351 |
| 12 | exp Forensic Microbiology/ | 58 |
| 13 | *Microbiological Examination/ | 11874 |
| 14 | *Molecular Diagnostic Techniques/ | 10881 |
| 15 | *Diagnostic Test/ | 16468 |
| 16 | *Specimen Handling/ | 676 |
| 17 | *Blood Sampling/ | 27354 |
| 18 | *Urine Sampling/ | 3200 |
| 19 | *Dissection/ | 8657 |
| 20 | *Blood Culture/ | 6686 |
| 21 | *Viral Load/ | 9046 |
| 22 | exp Pseudotyping/ | 881 |
| 23 | *Virus Culture/ | 4501 |
| 24 | *Virus Inactivation/ | 3325 |
| 25 | exp Xenodiagnosis/ | 532 |
| 26 | *High Temperature/ | 7796 |
| 27 | forensic microbiol*.ti,ab. | 68 |
| 28 | molecular technique*.ti,ab. | 15267 |
| 29 | molecular test*.ti,ab. | 22556 |
| 30 | specimen* handling.ti,ab. | 756 |
| 31 | (sear or sears or searing).ti,ab. | 1067 |
| 32 | cauter*.ti,ab. | 11142 |
| 33 | swabbing*.ti,ab. | 3072 |
| 34 | blood culture*.ti,ab. | 61656 |
| 35 | blood specimen*.ti,ab. | 11923 |
| 36 | urine specimen*.ti,ab. | 11517 |
| 37 | specimen collect*.ti,ab. | 6317 |
| 38 | sterile technique*.ti,ab. | 1362 |
| 39 | aseptic technique*.ti,ab. | 2584 |
| 40 | high temperature*.ti,ab. | 67674 |
| 41 | (heated adj1 blade*).ti,ab. | 9 |
| 42 | xenodiagnosis*.ti,ab. | 747 |
| 43 | 12 or 13 or 14 or 15 or 16 or 17 or 18 or 19 or 20 or 21 or 22 or 23 or 24 or 25 or 26 or 27 or 28 or 29 or 30 or 31 or 32 or 33 or 34 or 35 or 36 or 37 or 38 or 39 or 40 or 41 or 42 | 309508 |
| 44 | *Infection/ | 101024 |
| 45 | *Bacterial Infection/ | 70268 |
| 46 | *Bacteremia/ | 20110 |
| 47 | *Bacteriuria/ | 5069 |
| 48 | *Microflora/ | 23376 |
| 49 | contaminat*.ti,ab. | 338305 |
| 50 | infecti*.ti,ab. | 3059234 |
| 51 | pathogen*.ti,ab. | 1661433 |
| 52 | 44 or 45 or 46 or 47 or 48 or 49 or 50 or 51 | 4603596 |
| 53 | 11 and 43 and 52 | 6301 |
| 54 | (exp animal/ or exp invertebrate/ or nonhuman/ or animal experiment/ or animal tissue/ or animal model/ or exp plant/ or exp fungus/) not (exp human/ or human tissue/) | 9516554 |
| 55 | 53 not 54 | 5579 |
| 56 | limit 55 to english language | 5230 |
| 57 | limit 56 to embase | 2308 |

**Database:** **MEDLINE and Epub Ahead of Print, In-Process, In-Data-Review & Other Non-Indexed Citations, Daily and Versions (last upload update 27^th^ January 2026)**

Platform: Ovid

Publication Years: 1946 onwards

Date draft search was conducted on: 29^th^ January 2026

Limits: English Language Only

Filters: Human Studies Only Filter

Yield Results: 2520

Ovid Medline Persistent Search Link (via Monash University Library):

<https://access.ovid.com/custom/redirector/index.html?dest=https://go.openathens.net/redirector/www.monash.edu?url=http://ovidsp.ovid.com/ovidweb.cgi?T=JS&NEWS=N&PAGE=main&SHAREDSEARCHID=6zxggHClFnP2IJLSdQH7k4PrwYOSd7SfMCStIWT9MULSye2GJw1L8tlv6TNQshaTN>

**Search Strategy:**

| **#** | **Searches** | **Results** |
| --- | --- | --- |
| 1 | *Autopsy/ | 8952 |
| 2 | *Postmortem Changes/ | 4310 |
| 3 | *Cadaver/ | 3859 |
| 4 | autops*.ti,ab. | 85471 |
| 5 | (postmortem* or post-mortem*).ti,ab. | 74675 |
| 6 | (posthumous* or post-humous*).ti,ab. | 866 |
| 7 | cadaver*.ti,ab. | 74864 |
| 8 | necrops*.ti,ab. | 25430 |
| 9 | death.ti,ab. | 994893 |
| 10 | 1 or 2 or 3 or 4 or 5 or 6 or 7 or 8 or 9 | 1207520 |
| 11 | exp Forensic Microbiology/ | 6 |
| 12 | *Microbiological Techniques/ | 3588 |
| 13 | *Bacteriological Techniques/ | 14182 |
| 14 | *Molecular Diagnostic Techniques/ | 9035 |
| 15 | *Diagnostic Tests, Routine/ | 8831 |
| 16 | *Specimen Handling/ | 13799 |
| 17 | *Blood Specimen Collection/ | 6906 |
| 18 | *Urine Specimen Collection/ | 372 |
| 19 | *Dissection/ | 3871 |
| 20 | *Blood Culture/ | 1108 |
| 21 | *Viral Load/ | 7247 |
| 22 | exp Viral Pseudotyping/ | 51 |
| 23 | *Virus Cultivation/ | 4175 |
| 24 | *Virus Inactivation/ | 1230 |
| 25 | exp Xenodiagnosis/ | 122 |
| 26 | *Hot Temperature/ | 41237 |
| 27 | forensic microbiol*.ti,ab. | 62 |
| 28 | molecular technique*.ti,ab. | 12242 |
| 29 | molecular test*.ti,ab. | 12712 |
| 30 | specimen* handling.ti,ab. | 469 |
| 31 | (sear or sears or searing).ti,ab. | 681 |
| 32 | cauter*.ti,ab. | 5918 |
| 33 | swabbing*.ti,ab. | 2170 |
| 34 | blood culture*.ti,ab. | 34480 |
| 35 | blood specimen*.ti,ab. | 7181 |
| 36 | urine specimen*.ti,ab. | 7538 |
| 37 | specimen collect*.ti,ab. | 4043 |
| 38 | sterile technique*.ti,ab. | 699 |
| 39 | aseptic technique*.ti,ab. | 1362 |
| 40 | high temperature*.ti,ab. | 75334 |
| 41 | (heated adj1 blade*).ti,ab. | 7 |
| 42 | xenodiagnosis*.ti,ab. | 555 |
| 43 | 11 or 12 or 13 or 14 or 15 or 16 or 17 or 18 or 19 or 20 or 21 or 22 or 23 or 24 or 25 or 26 or 27 or 28 or 29 or 30 or 31 or 32 or 33 or 34 or 35 or 36 or 37 or 38 or 39 or 40 or 41 or 42 | 267110 |
| 44 | *Infections/ | 23545 |
| 45 | *Bacterial Infections/ | 53290 |
| 46 | *Bacteremia/ | 22931 |
| 47 | *Bacteriuria/ | 4804 |
| 48 | *Microbiota/ | 40136 |
| 49 | contaminat*.ti,ab. | 275499 |
| 50 | infecti*.ti,ab. | 2198729 |
| 51 | pathogen*.ti,ab. | 1266475 |
| 52 | 44 or 45 or 46 or 47 or 48 or 49 or 50 or 51 | 3385835 |
| 53 | 10 and 43 and 52 | 3132 |
| 54 | limit 53 to english language | 2893 |
| 55 | (exp animals/ or (rat or rats or mouse or mice or rodent* or swine or porcine or murine or sheep or lamb or lambs or pig or pigs or piglet or piglets or rabbit or rabbits or cat or cats or dog or dogs or cattle or bovine or monkey or monkeys or trout or marmoset or marmosets).ti.) not human*.sh. | 5601740 |
| 56 | 54 not 55 | 2520 |

**Database:** **Scopus**

Platform: Elsevier

Publication Years: 1922 onwards

Date draft search was conducted on: 29^th^ January 2026

Limits: English Language Only

Filters: Human Studies Only Filter

Yield Results: 8999

**Search Strategy:**

( ( INDEXTERMS ( Autopsy ) OR INDEXTERMS ( "Postmortem Changes" ) OR INDEXTERMS ( Cadaver ) OR TITLE-ABS ( autops* ) OR TITLE-ABS ( postmortem* OR post-mortem* ) OR TITLE-ABS ( posthumous* OR post-humous* ) OR TITLE-ABS ( cadaver* ) OR TITLE-ABS ( necrops* ) OR TITLE-ABS ( death ) ) AND ( INDEXTERMS ( "Forensic Microbiology" ) OR INDEXTERMS ( "Microbiological Techniques" ) OR INDEXTERMS ( "Bacteriological Techniques" ) OR INDEXTERMS ( "Molecular Diagnostic Techniques" ) OR INDEXTERMS ( "Diagnostic Tests, Routine" ) OR INDEXTERMS ( "Specimen Handling" ) OR INDEXTERMS ( "Blood Specimen Collection" ) OR INDEXTERMS ( "Urine Specimen Collection" ) OR INDEXTERMS ( Dissection ) OR INDEXTERMS ( "Blood Culture" ) OR INDEXTERMS ( "Viral Load" ) OR INDEXTERMS ( "Viral Pseudotyping" ) OR INDEXTERMS ( "Virus Cultivation" ) OR INDEXTERMS ( "Virus Inactivation" ) OR INDEXTERMS ( Xenodiagnosis ) OR INDEXTERMS ( "Hot Temperature" ) OR TITLE-ABS ( "forensic microbiol*" ) OR TITLE-ABS ( "molecular technique*" ) OR TITLE-ABS ( "molecular test*" ) OR TITLE-ABS ( "specimen* handling" ) OR TITLE-ABS ( sear OR sears OR searing ) OR TITLE-ABS ( cauter* ) OR TITLE-ABS ( swabbing* ) OR TITLE-ABS ( "blood culture*" ) OR TITLE-ABS ( "blood specimen*" ) OR TITLE-ABS ( "urine specimen*" ) OR TITLE-ABS ( "specimen collect*" ) OR TITLE-ABS ( "sterile technique*" ) OR TITLE-ABS ( "aseptic technique*" ) OR TITLE-ABS ( "high temperature*" ) OR TITLE-ABS ( heated W/1 blade* ) OR TITLE-ABS ( xenodiagnosis* ) ) AND ( INDEXTERMS ( Infections ) OR INDEXTERMS ( "Bacterial Infections" ) OR INDEXTERMS ( Bacteremia ) OR INDEXTERMS ( Bacteriuria ) OR INDEXTERMS ( Microbiota ) OR TITLE-ABS ( contaminat* ) OR TITLE-ABS ( infecti* ) OR TITLE-ABS ( pathogen* ) ) ) AND NOT ( ( ( INDEXTERMS ( animals ) OR TITLE ( rat OR rats OR mouse OR mice OR rodent* OR swine OR porcine OR murine OR sheep OR lamb OR lambs OR pig OR pigs OR piglet OR piglets OR rabbit OR rabbits OR cat OR cats OR dog OR dogs OR cattle OR bovine OR monkey OR monkeys OR trout OR marmoset OR marmosets ) ) NOT INDEXTERMS ( human* ) ) ) AND ( LIMIT-TO ( LANGUAGE , "English" ) )
